# Online education isn’t the best choice: evidence-based medical education in the post-epidemic era—a cross-sectional study

**DOI:** 10.1186/s12909-023-04746-8

**Published:** 2023-10-10

**Authors:** Yi Zhang, Jiarui Liu, Jun Liang, Jie Lang, Lijia Zhang, Mingwen Tang, Xinyu Chen, Yan Xie, Jianlin Zhang, Liyu Su, Xin Wang

**Affiliations:** 1 Department of Hygiene Toxicology, School of Public Health, Zunyi Medical University, Zunyi, Guizhou 563000 China; 2 Key Laboratory of Maternal & Child Health and Exposure Science of Guizhou Higher Education Institutes, Zunyi Medical University, Zunyi, Guizhou 563000 China; 3 School of Public Health, Guizhou Medical University, No. 6, Ankang Avenue, Machang Town, Guian New District, Zunyi, Guizhou Province China; 4 School of Chemical Engineering and Light Industry, Guangdong Industry Polytechnic, Guangzhou, Guangdong 510300 China; 5 Enrollment and Employment Office, Zunyi Medical University, Zunyi, Guizhou 563099 China; 6 Department of Orthopaedic Surgery, Affiliated Hospital of Zunyi Medical University, Zunyi, Guizhou 563003 China; 7 Guizhou Provincial Key Laboratory of Medicinal Biotechnology in Colleges and Universities, Zunyi Medical University, Zunyi, Guizhou 563000 China

**Keywords:** Evidence-based medicine, Online education, Blended education, Offline education, Medical schools

## Abstract

**Background:**

The COVID-19 pandemic led many educational institutions to shift to online courses, making blended education a significant trend in teaching. We examined the effectiveness of blended learning in an evidence-based medicine course.

**Methods:**

We compared the examination scores of a blended learning group, an online only group, and a traditional offline group and conducted a questionnaire survey on students’ preferences for different learning modes and the reasons for their preferences. A total of 2100 undergraduate students in clinical medicine were included in this cross-sectional study. Examination results were collected, and questionnaires were administered to the study participants. We compared the mean scores and exam pass rates of the three teaching groups using ANOVA and c^2^test for multiple comparisons.

**Results:**

The blended group’s exam scores and pass rate were significantly higher than those of the offline and online groups. Furthermore, 71.6% preferred the blended teaching mode. In the survey on " learning effectiveness”, the majority of the students believed that blended education could better enhance the initiative of learning, the interest of the course, the pertinence of the learning content, the comprehension of evidence-based medical thinking, and the basic skills of evidence-based practice. Subsequently, in a questionnaire administered to a blended group of students, their foremost reason for liking online instruction was ‘flexible in time and space’ (99%), followed by ‘can be viewed repeatedly, facilitating a better understanding of knowledge points’ (98%). Their foremost reason for liking offline teaching was ‘helps to create a good learning atmosphere’ (97%), followed by ‘teachers can control students’ learning status in real time’ (89%).

**Conclusions:**

This study explored the effectiveness of learning in evidence-based medicine courses by comparing the learning outcomes and personal perceptions of three different teaching modes. This is the first cross-sectional study in which three different teaching models are compared and discussed in an evidence-based medicine course. We also elaborate on the specific instructional protocols for each model. This study shows that using a blended education approach in evidence-based medicine courses can improve students’ learning motivation, autonomy, and satisfaction. It also enhances instructional efficiency, thereby improving students’ understanding of the course content.

**Supplementary Information:**

The online version contains supplementary material available at 10.1186/s12909-023-04746-8.

## Background

The emergence of the coronavirus disease 2019 (COVID-19) posed a great challenge to public health security worldwide [1, 2]. The World Health Organization declared it a global pandemic in March 2020 [3]. Consequently, governments implemented lockdowns, travel restrictions, and quarantines to control the spread of the pandemic [4, 5]. These restrictions had a dramatic impact on pre-existing teaching and learning models in higher education institutions worldwide. Could the COVID-19 pandemic be an opportunity to rethink the understanding of higher education?

Since the threat of COVID-19 began, educational institutions globally started online classes by actively preparing for teaching online and suspending offline teaching [6]. The impact of this shift is particularly evident for medical schools that focus on theoretical knowledge and practical skills. Medical courses must combine theory and practice simultaneously to impart the solid theoretical knowledge necessary to apply practical knowledge, skills, and techniques to clinical and research practice. Medical schools had to thoroughly explore reasonable teaching methods to determine how best to teach ‘hands-on’ medicine while protecting students from the deadly contagion. Online education suddenly shifted from a supplementary teaching tool to the primary education mode; this shift included medical education [7, 8], which further increased educators’ interest. Is online education the best option for higher medical schools? A systematic review revealed that online medical teaching during COVID-19 was effective [9]. Another study showed that students found online instruction a great time saver [10]. Angie et al. [11] also confirmed this, with the majority of students finding online instruction more convenient and comfortable, allowing flexibility in the pace and rhythm of learning. Similarly, Ashour et al.’s [12] online survey revealed that the vast educators believed online education can be widely adopted and can make a greater contribution to higher education in the future. However, every coin has two sides. Many educators and scholars feared online education would disrupt traditional classrooms and eventually end on-site schooling [13]. Others argued that online education could not achieve the same outcomes as face-to-face education and should only be used to complement in-person classroom teaching [14]. In line with this argument, a study by Wang et al. [15] found that students were not satisfied with online teaching, believing there is less discussion and interaction compared to traditional offline teaching. Another study by Aslam et al. [16] concluded that in the field of medical education, moving from traditional face-to-face teaching to online teaching would take more time and experience. There are also other disparate views. Stevens et al. [17] reviewed 91 comparative studies between 2000 and 2020, finding that 41% of the studies determined that online instruction was associated with better learning outcomes; however, another 41% reported no significant difference between online and face-to-face learning. Holloway et al. [18] similarly concluded that student performance in critical thinking skills did not differ significantly between online and face-to-face learning modes. While every country has made tremendous progress in controlling COVID-19, higher education has not yet been restored to pre-pandemic conditions. Educators have been grappling with whether medical schools should return entirely to offline, face-to-face teaching or use the opportunity to build a future-oriented teaching model incorporating the advantages of online teaching.

Evidence-based medicine (EBM) is a compulsory basic course of clinical medicine, preventive medicine, and most other medical specialities in medical colleges. Learning EBM helps prepare students to align the best research evidence and clinical expertise with patients’ unique values and personal circumstances [19, 20]. Many countries, including China, teach EBM in their medical colleges and universities because studies show that it can improve medical students’ critical thinking, clinical problem-solving, innovation, learning motivation, attitudes, knowledge, and self-reported critical appraisal skills [21].

With the world entering the post-epidemic era, blended teaching as well as learning both online and offline has become a new trend [22–24]. A recent meta-analysis reviewed medical education studies from 1990 to 2019, comparing blended and face-to-face learning; it found that blended teaching improves student learning [25]. Similarly, Kang et al. concluded that compared to traditional teaching, blended teaching methods improved students’ knowledge, problem-solving skills and learning satisfaction in public health courses [26]. However, effective blended teaching and learning requires time and effort from educators and learners to make specific adjustments [27]. The design of blended instruction is more demanding because of the multidisciplinary and practical nature of EBM. To our knowledge, no studies have systematically elaborated on the specific outcomes of blended teaching in EBM courses.

As a new model combining online and offline teaching, most schools view online teaching as a simple complement to offline teaching, making no efforts to improve it according to the actual teaching situation [25]. Consequently, there remains a need to study the current situation and impact of three different models of online, offline, and blended teaching on medical students’ learning. However, no studies thus far have compared the specific learning effects of the three different teaching modes in EBM courses.

Using the teaching of an EBM course as an example, this study aimed to explore the exam results of different teaching models in a new age context, as well as students’ personal preferences and perceptions of different teaching models. To our knowledge, this is the first cross-sectional study in which three different teaching models are compared and discussed in an EBM course. We also elaborate on the specific instructional protocols for each model. Our study is the first to systematically describe the specific effects of blended instruction in an EBM course. It informs the implementation of the EBM curriculum and the development of future teaching models, promoting new information technologies for the whole process of education and teaching.

## Methods

### Participants

The participants in this cross-sectional study were 2,100 undergraduate students (aged 20–23) in clinical medicine at Zunyi Medical University (ZMU) in Zunyi, Guizhou, China, who took an EBM course during the 2018–2021 academic year. We excluded students who do not take exams and who miss classes and fail to complete the course. We analysed 700 students in each of the three groups using simple random sampling. They are traditional offline (academic year 2018–2019), purely online (academic year 2019–2020), and blended (academic year 2020–2021). The same 12 teachers at ZMU taught the courses and sections.

### Teaching content and lesson schedule

ZMU’s EBM course focuses on teaching five skills. *Evidence retrieval* means identifying and linking evidence sources using a specific clinical problem as a pivot, which involves selecting databases, identifying search terms, developing search strategies, searching databases, and presenting search results for different clinical questions or study designs. *Evidence assessment* means evaluating evidence for truthfulness and accuracy, significance, and applicability to specific clinical issues, including assessing relevant reports’ methodological quality and validity and the quality and validity of the evidence reported. *Evidence comparison* means systematically synthesising homogenous studies (meta-analyses). *Evidence application* means practicing EBM in the context of clinical practice, which requires a comprehensive understanding of the EBM research method. Finally, *evidence development* means conducting investigations to produce empirical evidence. These five skills provide a foundation for undergraduate students to build their EBM clinical practice abilities based on evidence.

The undergraduate EBM course required 36 h of classes and used the textbook *Evidence-based Medicine* [29]. All three groups followed the same syllabus, and the lecture duration was the same.

#### Traditional offline

Senior professors conducted traditional offline, face-to-face teaching, primarily lectures presenting introductory and general content and instruction for self-study.

#### Purely online

During the COVID-19 pandemic, the same teachers switched to online courses to protect the safety of the teachers and students. ZMU’s online teaching platforms are the China University Massive Open Online Course (MOOC) and Superstar Learning. Both platforms offer educational video resources and features like quizzes and learning statistics. The same senior professors taught the online courses, selecting various options from the platforms according to their teaching style but covering the same content as the offline course [15, 28].

#### Blended

Finally, the same senior professors taught the blended EBM course combining traditional offline lectures and virtual classes using two online teaching platforms. The EBM course syllabus was divided into two parts: online teaching and offline classroom teaching (case-based learning or problem-based learning). The online teaching portion emphasised training in evidence-based thinking and research competencies. The offline education portion emphasised disseminating knowledge-based content. Table 1 shows the specific implementation of the blended teaching mode.

**Table 1:** Evidence-based medicine blended teaching reform programme

Course content	Class hour	Teaching mode	Learning objectives
Chapter 1. General introduction to evidence-based medicine	2	Online and Offline	Evidence retrieval
Chapter 2. Finding and raising questions in clinical practice	2	Online	
Chapter 3. Classification, classification, and recommendation of evidence	2	Online	
Chapter 4. Evidence sourcing and retrieval	2	Offline	
Chapter 5. Overview of evidence evaluation in clinical studies	2	Online	Evidence assessment
Chapter 6. Overview of evidence evaluation in clinical studies	2	Online	
Chapter 7. Applying statistical methods in evidence-based medicine	2	Offline	
Chapter 8. Systematic evaluation	4	Online and Offline	Evidence comparison
Chapter 9. Meta-analysis	4	Online and Offline	
Chapter 10. Evaluating and applying research evidence on etiology and adverse reactions	2	Online and Offline	Learn to apply evidence
Chapter 11. Evaluating and applying evidence in diagnostic studies	2	Online and Offline	
Chapter 12. Evaluating and applying therapeutic research evidence	2	Online and Offline	
Chapter 13. Evaluating and applying prognostic research evidence	2	Online and Offline	
Chapter 14. Evaluating and applying clinical guidelines	2	Online	Learn to develop evidence empirically
Chapter 15. Evaluating and applying clinical economic evidence	2	Offline	
Chapter 16. Teaching evidence-based medicine		Offline	
Chapter 17. Clinical trial transparency	2	Online	

The following examples describe the blended teaching mode using the China University MOOC and Superstar Learning platforms.

#### Class preparation (Online)

The students logged onto the EBM course on the China University MOOC website (https://www.icourse163.org/). They answered a set of questions before watching the video module. Then, they watched the chapters’ instructional videos online and completed the instructors’ assigned tasks, referencing the textbook. Since studying with questions in mind can dramatically improve learning efficiency, the teachers encouraged the students to approach the online modules to resolve issues they might have encountered in their independent study. The students consulted with classroom teachers or classmates offline if they needed assistance.

#### Classroom study (Offline)

Once the students completed the online class preview module, the teacher used the results to refine the classroom lecture to address areas where the students required additional knowledge and clarification. They encouraged the students to ask questions, engage in discussions, and reflect deeply on the answers.

#### Independent study

After the classroom lesson, the students logged into the teachers’ homework database on the Superstar Learning database and completed the tasks and examinations. This enabled the teachers to gauge the students’ knowledge mastery and helped the students consolidate their online–offline knowledge.

### Outcome measurements

ZMU’s School of Public Health assessed the students in all three groups (traditional offline, purely online, and blended) on the same core content of the syllabus. The exams were closed-book written exams evaluating the students’ mastery of theoretical and practical knowledge. All three groups of test questions were of the same difficulty level.

### Questionnaire for the blended group

In order to further explore which mode was preferred by the students, a questionnaire was administered only to the blended group of students who had adopted both the three modes. We developed and administered a questionnaire survey to ask which learning methods they preferred and why to thoroughly analyse the three modes’ effectiveness and the students’ perspectives. We explained that (1) completing the questionnaire was optional; (2) their decision to complete it (or not) would not affect their course grade in any way; and (3) their questionnaire would be completely anonymous. We made the questionnaire available online after the course ended. The questionnaire covered these main areas: which of the three modes they preferred (single choice); which they considered the most effective (single choice); and their personal feelings about each of the three modes (outcomes were assessed using a three-level likert scale, option “1” indicates agree, option “2” indicates average, and option “3” indicates disagree) A total of 683 students (97.6%) completed the questionnaire.

### Statistical analysis

We compared the mean scores and exam pass rates of the three teaching groups using ANOVA and c^2^test for multiple comparisons. All *p*-values were two-sided, with *p* < 0.05 considered statistically significant. We performed all statistical analyses using IBM SPSS Statistics for Windows, Version 23.0 (Armonk, NY: IBM Corp.).

## Results

### Participant characteristics

All the participants in the study fell within the age range of 20 to 23. To determine whether there were differences in the baseline academic achievement between the offline, online, and blended groups, we analysed the participants’ results from their National College Entrance Examinations. The analysis revealed that the average grades of the three groups of students in the offline, online and blended groups in the National College Entrance Examinations were 532 (± 72), 530 (± 73) and 533 (± 64) respectively. We found no statistical difference in the admission scores between the three groups (*p* > 0.05).

### Assessment results comparison for the three modes

**Fig. 1:**
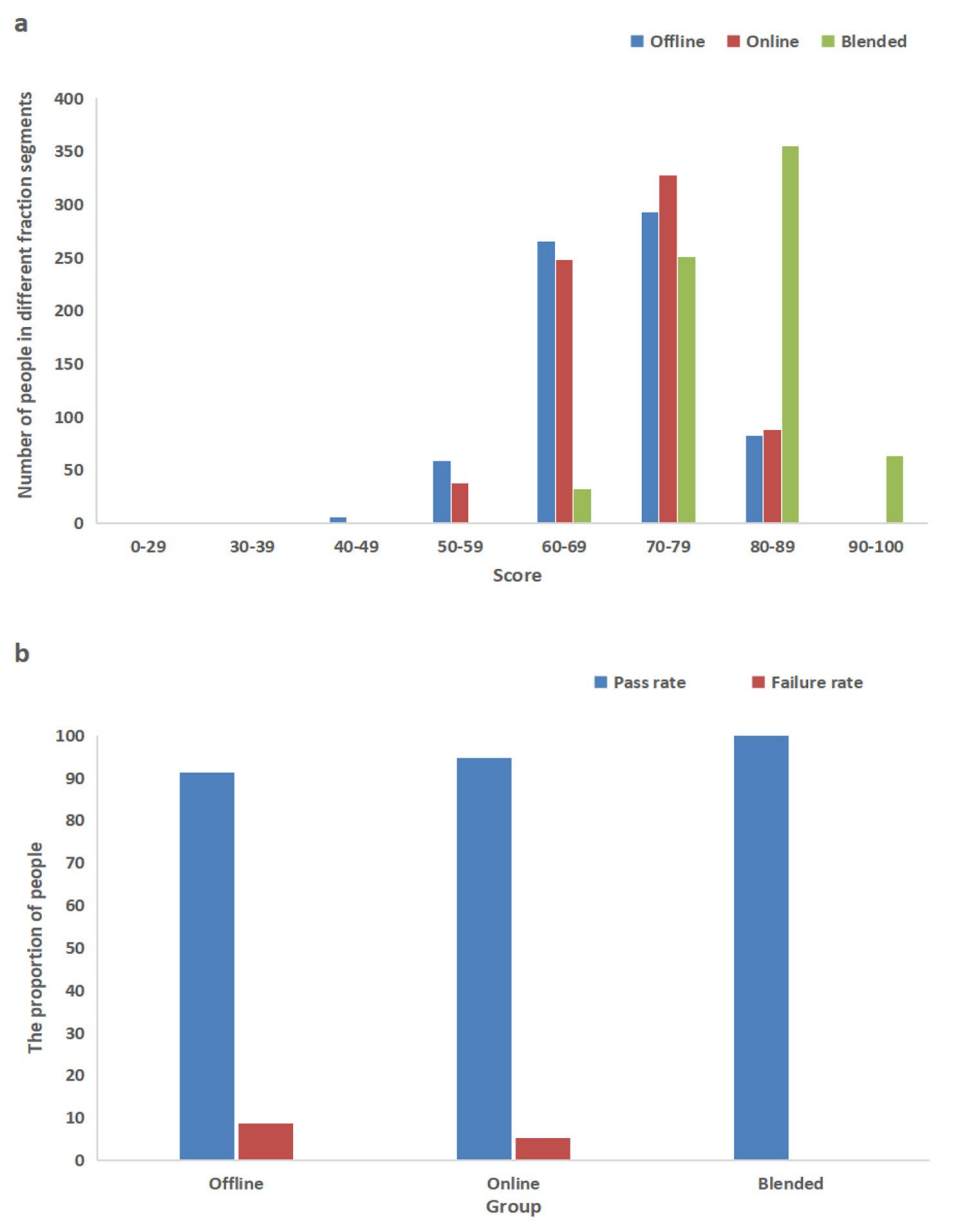
Comparison of the assessment results of the three different teaching modes

We compared the assessment scores of the three groups (traditional offline, purely online, and blended), including their theoretical scores and exam pass rates. The theoretical score was the student’s final exam grade. The assessment of regular grades included ‘chapter quizzes’, ‘completing the online and offline assignments after each chapter’, ‘presentation in class discussions’, and ‘attendance’. The blended group’s exam scores were significantly higher than those of the offline and online groups. As seen in Table 2, there were significant differences in the performances of the three modes (*p* < 0.001). Additionally, the difference between the blended group and the online group was statistically significant, *P* < 0.001. Comparing the scores revealed that significantly more students in the blended group scored 70–79 and 80–89 points than in the offline and online groups, and the only students with scores of 90–100 were in the blended group (Fig. 1a). The blended group had a significantly higher pass rate and lower failure rate (Fig. 1b).

**Table 2:** Assessment results of the three different teaching modes

Variables	Blended group	Online group	Offline group	*P*
Average test score	69.91 ± 10.75*	57.72 ± 12.16	59.21 ± 10.96	< 0.001^α^
Examination pass rate (%)	100*	94.7	91.3	< 0.001^b^

*The difference was statistically significant compared with the online group, *P* < 0.001

^α^*p*-value was calculated via ANOVA;^b^*p*-value was calculated via c^2^test

### Survey results

**Fig. 2:**
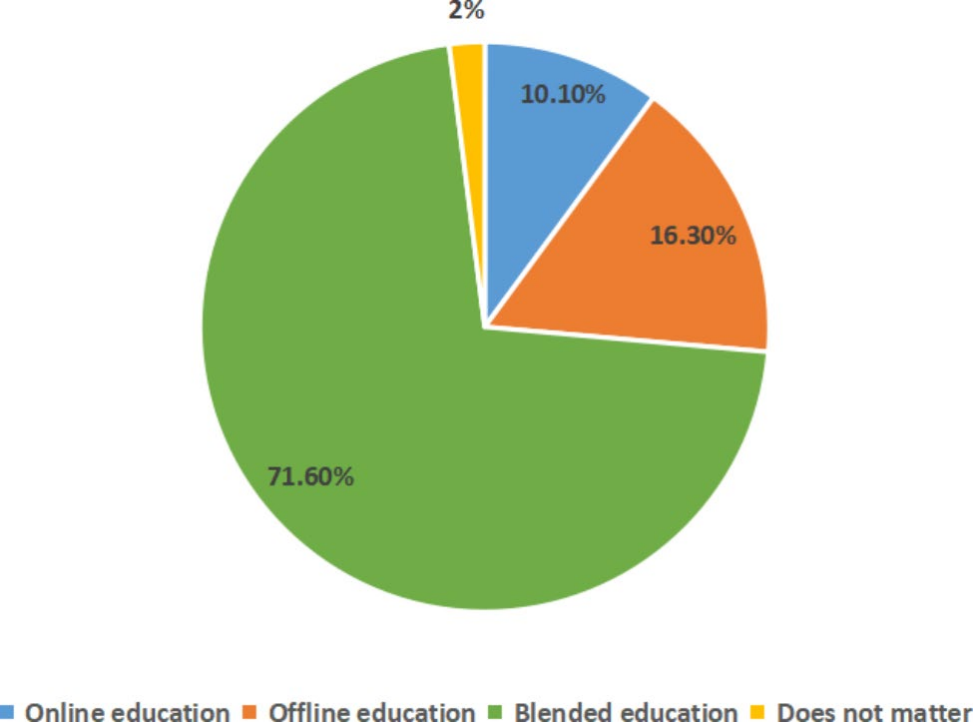
Students’ favorite teaching mode

The questionnaire survey had three categories of questions. The first category concerned which teaching mode the students preferred. Overall, 71.6% preferred the blended teaching mode; they believed they benefited more from it than the online or offline teaching modes (Fig. 2).

**Fig. 3:**
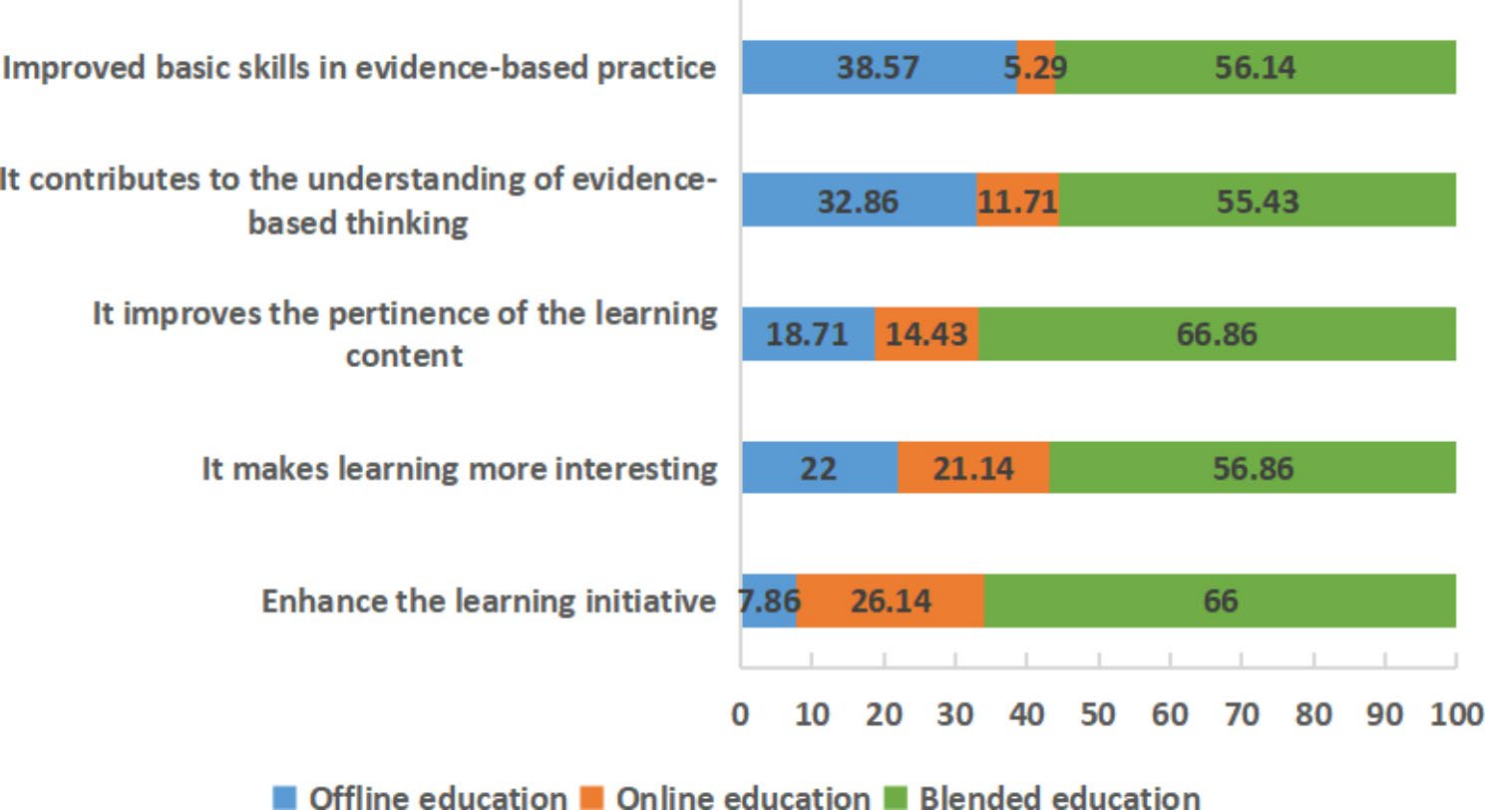
A survey of the “learning” effectiveness

The second category concerned which teaching mode they considered most effective. Most of the students believed that blended education could better enhance the initiative of learning. Their ratings for ‘course interest’, ‘content relevance’, ‘comprehension of evidence-based medical thinking’, and ‘basic skills for evidence-based practice’ were significantly higher for the blended mode than for the offline or online teaching modes (Fig. 3).

**Fig. 4:**
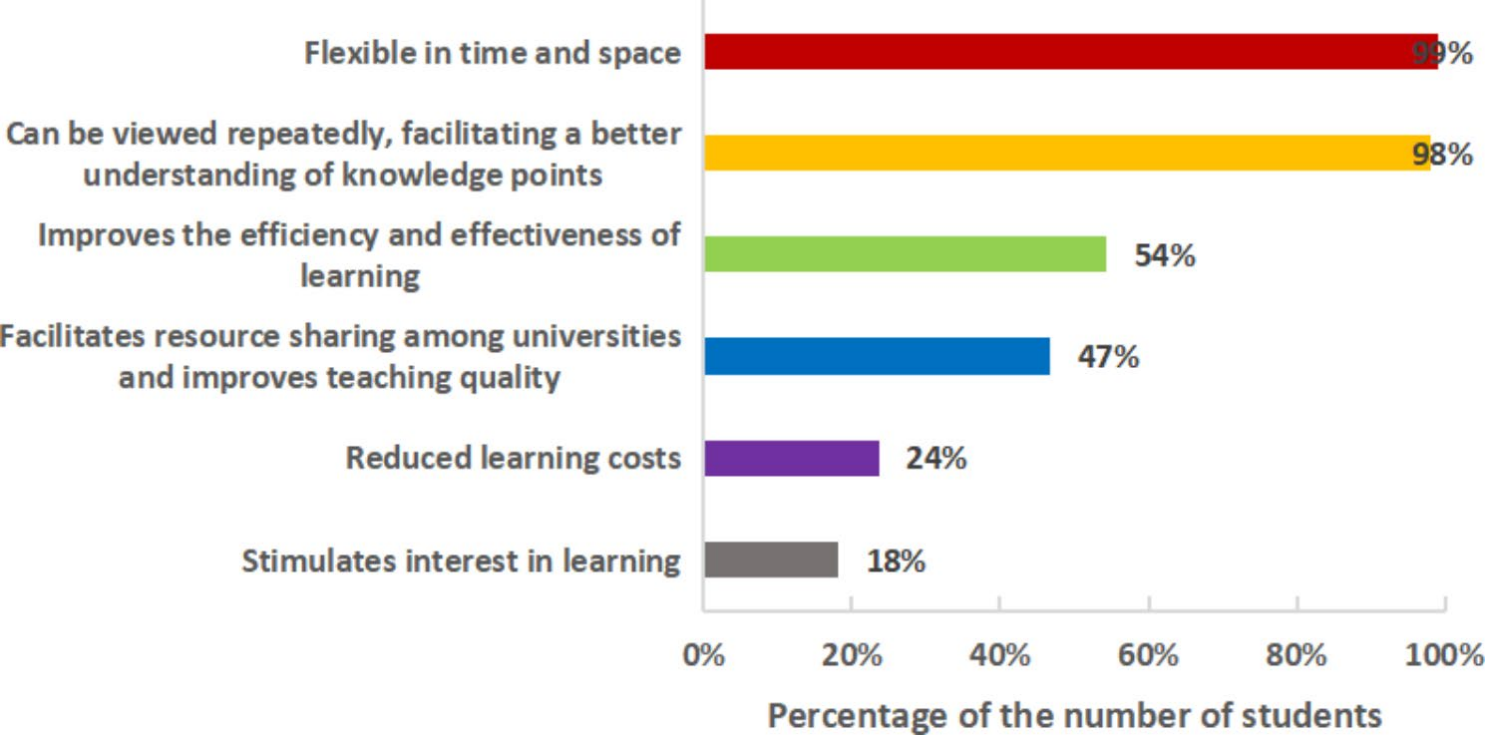
Advantages of online courses

The third category concerned the students’ feelings about each of the different modes. Their foremost reason for liking online instruction was ‘flexible in time and space’ (99%), followed by ‘can be viewed repeatedly, facilitating a better understanding of knowledge points’ (98%); ‘improves the efficiency and effectiveness of learning’ (54%); ‘facilitates resource sharing among universities and improves teaching quality’ (47%); ‘reduces learning costs’ (24%); and ‘stimulates interest in learning’ (18%) (Fig. 4).

**Fig. 5:**
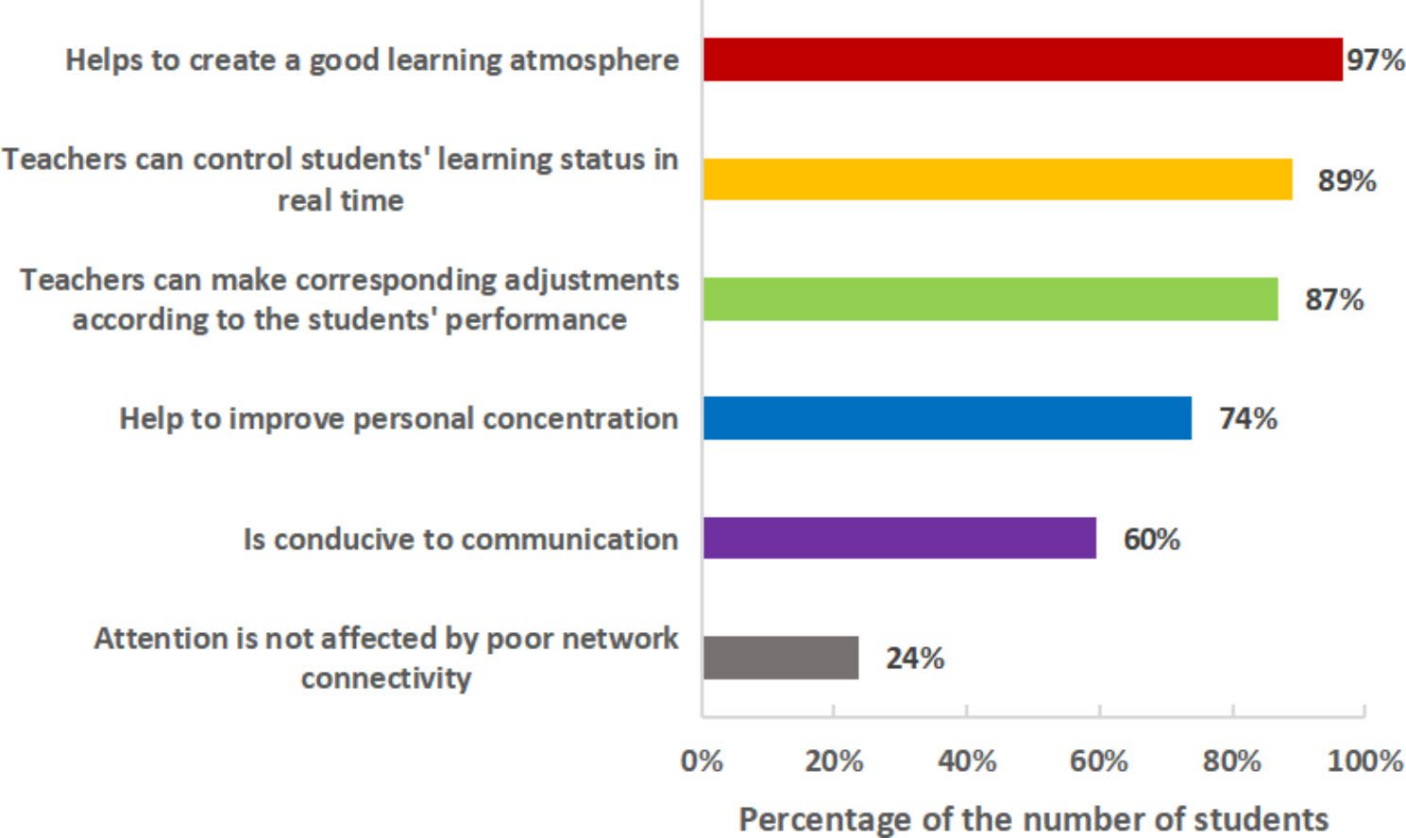
Advantages of offline courses

The students’ foremost reason for liking offline teaching was ‘helps to create a good learning atmosphere’ (97%), followed by ‘teachers can control students’ learning status in real time’ (89%). Figure 5 shows the other reasons.

## Discussion

Recent advances in information technology (IT), especially the internet, smartphones, and conferencing software, have inspired educators to explore new teaching modes that integrate online technology to organise the classroom [25, 29, 30]. The COVID-19 pandemic accelerated the shift to online courses, and many now recognise that blended teaching has enriched the educational format. Medical educators indicate that blended learning allows for clinical practice activities, clinical thinking discussions, and role plays through offline learning, which can be complemented via online learning to review, consolidate, and extend the knowledge gained in offline courses [27]. Blended learning has advantages over traditional learning and can improve the quality of medical education as a promising educational programme [31]. In average test scores and pass rates for the EBM courses, the blended group outperformed the other two groups in both learning outcomes. The results showed that students in our university’s EBM course strongly preferred the blended teaching mode to purely online or offline teaching. Most students said that blended teaching enabled them to grasp the course’s knowledge points more effectively than the other teaching modes. Students’ ratings for ‘course interest’, ‘content relevance’, ‘comprehension of evidence-based medical thinking’, and ‘basic skills for evidence-based practice’ were significantly higher for the blended mode than for the offline or online teaching modes.

Research on how IT has impacted education, especially following the COVID-19 pandemic, suggests that the future of education will include teaching in some offline–online hybrid forms [32]. We found that students considered online teaching ‘flexible’, ‘convenient’, ‘efficient,’ and ‘effective,’ largely because it does not restrict teaching to specific times, locations, or frequency. This aligned with others’ findings that students appreciate the flexibility of online education over time and space [33]. Online and blended teaching can maximise limited resources (e.g., classroom space) and broaden the reach while still allowing teacher–student interaction. Nearly all the students in this study said the main advantages of online learning were its flexibility and autonomy (99%), and its content could be viewed repeatedly (98%). Multiple studies have shown that students appreciate how online learning’s flexibility meets their individual learning abilities and preferences, enabling them to revisit whole or parts of lessons whenever and wherever they choose using their computers, tablet, or smartphone, increasing the efficiency and effectiveness of learning [34–36]. In addition, China’s online teaching platforms offer high-quality courses available nationally, which considerably improves the problem of unequal distribution of educational resources. It also reduces the distance between teaching and learning and between teachers and students. Students can access free presentations, class materials, and exercises from online courses, reducing learning costs [37]. However, online education is no panacea. By comparing the two models, Chi-Chung et al. [38] noted that online instruction was much less effective than offline instruction. Similarly, Christian et al. [39] noted that online education mitigated some of the negative effects that occurred because of the COVID-19 embargo, and that although some students showed a positive attitude toward online instruction, most students preferred the traditional face-to-face instruction model. Online teaching does not work for classes that require hands-on or team-based interaction. Online videotelephony and videoconferencing applications (e.g., Zoom) expand classes’ reach but can make it difficult for students to interact meaningfully with teachers, classmates, or—for medical students—patients [40]. For example, one recent study reported that students with greater exposure to online teaching exhibited less engagement in collaborative learning activities [41]. During COVID-19, student engagement during online learning was low due to reduced interpersonal interactions, that is, student-student and student-instructor interactions [42]. Ryan et al. [43] argued that while online learning may be a valuable solution to mitigate the spread of the virus during the COVID-19 outbreak, it is not the best option for medical education; instead a blended format combining online and offline could be considered.

Our research showed that traditional offline education was more conducive to face-to-face teacher–student and student–student interactions and created a good learning atmosphere. Teachers can more easily monitor students’ reactions in person and gauge how many and which students might be struggling with the material, which can lead to more targeted learning. In-person classroom responses are easier to parse than a screenful of tiny online faces, so teachers can observe students’ mastery levels in real time and adjust accordingly [44]. Furthermore, some researchers have reported that in traditional offline classes, teachers can develop antagonistic, condescending, power-oppressive relationships with students, leading to a tense atmosphere that discourages shy students from answering teachers’ questions [37]. The students in our study reported that traditional offline learning created a good learning atmosphere that improved their concentration. However, offline education has no advantages over online education in the areas of online resources and statistical analyses [45]. For example, when students complete offline homework assignments, the teachers usually must manually calculate the grades and track each student’s task completion. The manual calculation and entry processes can be time-consuming, inefficient, and prone to errors. Online teaching platforms streamline the process of calculating and tracking grades and task completion, improving efficiency and reducing errors. In addition, the effectiveness of teaching methods is better reflected in the blended teaching which combined with online and offline modes. It is well known that teaching methods may also have a certain impact on the teaching effect. The course content of evidence-based medicine itself is based on a variety of clinical research questions and clinical case studies that unfold. So in fact, both traditional offline group and blended group had taking implementing case-based learning or problem-based learning. However, the teaching effect of offline group is not very well. This could be due to didactic explanations making it difficult for offline group students to understand abstract points, as a result, they are not interested in the case analysis. Blended mode of teaching take advantage of the vividness of the online course, which in turn makes the case problems, which are difficult to understand, more intuitive. It also combines the advantages of offline teaching, where teachers can put forward questions based on each student’s performance. The students can be put into the role of the case better, which helps them to understand the course content better.

Our study still had some limitations. First, our questionnaire respondents were all taken from one cohort of clinical medical students who had experienced blended teaching, and the findings represented their subjective feedback on the different teaching modes. The other two groups were not investigated, which would have reduced the reliability of the response. Second, the participants in the blended group scored higher on the exam than those in the online and offline groups, which probably influenced their positive views on blended teaching. Furthermore, we obtained our results through a transversal comparison of two cohorts from different years. Future studies should broaden the participant pool to include a larger number of students in other academic years and from other medical schools to remove confounding variables. Randomised controlled comparisons using the same year cohort might yield results that differ from current results.

## Conclusions

This paper explains in detail the differences between online, offline, and blended education using the example of EBM. In line with other studies, our research found pros and cons for online and offline teaching. Online education, which is currently the most popular, is not the best choice; particularly we found that students prefer blended teaching to purely offline or online classes. Additionally, blended instruction can combine the two teaching methods, using their respective strengths to maximum advantage; the flexibility, portability, repeatability, and efficiency of online learning complements the positive learning atmosphere, real-time monitoring and adaptation, and immediacy of offline learning to create a ‘one plus one is greater than two’ instructional effect. We propose that the ideal educational model is blended, with the ratio of online to offline teaching tailored according to the nature of the course and its content. Courses with complex and detailed content would likely have more online components to allow students to revisit the lessons as often as necessary; in contrast, courses requiring hands-on practice would likely have more offline content. Overall, our study found that EBM students preferred blended learning that combined the advantages of online and offline. We recommend that universities—including medical schools—expand their use of blended teaching.

### Electronic supplementary material

Below is the link to the electronic supplementary material.

Supplementary Material 1

## Data Availability

All data generated or analyzed during this study are included in this published article.

## Abbreviations

EBM: Evidence-based medicine
IT: Information technology
MOOC: Massive Open Online Course
