# Supplementary material for: Online education isn’t the best choice: evidence-based medical education in the post-epidemic era—a cross-sectional study
Source: BMC Med Educ. 2023 Oct 10;23:744. doi: 10.1186/s12909-023-04746-8 (PMC10563228; doi:10.1186/s12909-023-04746-8)
Supplement: Supplementary file 1 — Supplementary Material 1 [file 12909_2023_4746_MOESM1_ESM.docx]

Questionnaire survey on teaching model of the ‘evidence-based medicine course’

Hello! It is my pleasure to invite you to complete this questionnaire. To better understand the effect of learning and personal feelings after completing this course, please read the questions carefully, and choose the answer that best matches your idea. Please read the questions and choose your answer. The questionnaire is anonymous, there are no right or wrong answers, and no personal information about you will appear, so please feel free to fill it out.

Thank you for your support!

1. Your favourite teaching model (single choice)

A. Online education

B. Offline education

C. Blended education

D. None/Uncertain

Evaluation of the learning effect of different teaching modes (single choice)

2. Which model do you think is better for improving basic skills in evidence-based practice？

A. Online education

B. Offline education

C. Blended education

3. Which model do you think is helpful in understanding evidence-based thinking？

A. Online education

B. Offline education

C. Blended education

4. Which model do you think improves the pertinence of the learning content？

A. Online education

B. Offline education

C. Blended education

5. Which model do you think improves the interest of learning?

A. Online education

B. Offline education

C. Blended education

6. Which model do you think enhances the learning initiative？

A. Online education

B. Offline education

C. Blended education

Please provide your opinion about online education. The opinions of the evaluation were categorized into three levels, using statements on a Likert scale score (1 = disagree, 2 = indifferent/uncertain, 3 = agree).

1. Facilitates resource sharing among universities and improves teaching quality

1-Do not agree

2-indifferent / uncertain

3-Agreed

2. Flexible in time and space

1-Do not agree

2-indifferent / uncertain

3-Agreed

3. Can be viewed repeatedly, facilitating a better understanding of knowledge points

1-Do not agree

2-indifferent / uncertain

3-Agreed

4. Improves the efficiency and effectiveness of learning

1-Do not agree

2-indifferent/uncertain

3-Agreed

5. Reduced learning costs

1-Do not agree

2-Indifferent/uncertain

3-Agreed

6. Stimulates interest in learning.

1-Do not agree

2-indifferent / uncertain

3-Agreed

Your opinion about offline education. The opinions of the evaluation were categorized into three levels, using statements on a Likert scale (1 = disagree, 2 = indifferent/uncertain, 3 = agree).

1. Teachers can control students’ learning status in real time.

1-Do not agree

2-Indifferent/uncertain

3-Agreed

2. Helps to create a good learning atmosphere

1-Do not agree

2-Indifferent/uncertain

3-Agreed

3. Teachers can make corresponding adjustments according to the students’ performance

1-Do not agree

2-Indifferent/uncertain

3-Agreed

4. Help to improve personal concentration

1-Do not agree

2-Indifferent/uncertain

3-Agreed

5. Is conducive to communication

1-Do not agree

2-Indifferent/uncertain

3-Agreed

6. Attention is not affected by poor network connectivity

1-Do not agree

2-Indifferent/uncertain

3-Agreed
